# MYD88^L265P^ and MYD88^other^ variants show different molecular characteristics and prognostic significance in diffuse large B-cell lymphoma

**DOI:** 10.1007/s00432-023-04714-1

**Published:** 2023-04-24

**Authors:** Yan Qin, Tian Qiu, Zucheng Xie, Xinrui Chen, Peng Liu, Jianliang Yang, Xiaohui He, Lin Gui, Shengyu Zhou, Hongxin Jiang, Changgong Zhang, Sheng Yang, Le Tang, Yuankai Shi

**Affiliations:** 1 Department of Medical Oncology, National Cancer Center/National Clinical Research Center for Cancer/Cancer Hospital, Chinese Academy of Medical Sciences & Peking Union Medical College, Beijing Key Laboratory of Clinical Study on Anticancer Molecular Targeted Drugs, Beijing, 100021 China; 2 Department of Pathology, National Cancer Center/National Clinical Research Center for Cancer/Cancer Hospital, Chinese Academy of Medical Sciences & Peking Union Medical College, Beijing, 100021 China; 3 Department of Medical Oncology, Suzhou Municipal Hospital, Affiliated Suzhou Hospital of Nanjing Medical University, Suzhou, 215001 China

**Keywords:** DLBCL, MYD88 variation, Prognosis

## Abstract

**Purpose:**

This study aims to investigate the clinical and molecular differences between diffuse large B-cell lymphoma (DLBCL) patients with MYD88^L265P^ and MYD88^other^.

**Methods:**

DLBCL patients with MYD88 variations were collected from the Cancer Hospital, Chinese Academy of Medical Sciences & Peking Union Medical College (CHCAMS), and Suzhou Municipal Hospital from February 6th, 2007 to May 20th, 2022. Clinicopathological parameters and treatment outcomes between MYD88^L265P^ and MYD88^other^ were investigated.

**Results:**

A total of 132 patients with MYD88 variations from a cohort of 475 DLBCL patients were included, among which, 78 were MYD88^L265P^, while 54 were MYD88^other^. MYD88^L265P^ was more common in non-GCB subtype than MYD88^other^ (83% vs. 60%, *P* = 0.004). Besides, MYD88^L265P^ was significantly related to higher proportion of testicle/ central nervous system involvement (31% vs. 6%, *P* < 0.001), PIM1 mutation (71% vs. 39%, *P* < 0.001), and PIM1 hypermutation (28% vs. 11%, *P* = 0.018), compared with MYD88^other^. Compared with MYD88^L265P^, MYD88^other^ were more likely to have higher percentage of advanced stage (60% vs. 42%, *P* = 0.044), extranodal site ≥ 2 (45% vs. 28%, *P* = 0.044), elevated LDH (55% vs. 35%, *P* = 0.033), positive CD10 expression (36% vs. 16%, *P* = 0.009), BCL-6 translocation (20% vs. 8%, *P* = 0.033), and NOTCH pathway gene alteration (24% vs. 13%, *P* = 0.040). In non-GCB DLBCL subtype, patients with MYD88^other^ were significantly associated with worse progression free survival (PFS) than those with MYD88^L265P^ when treated initially with R-CHOP/R-CHOP-like regimen (*P* = 0.010).

**Conclusion:**

The findings of this study indicate that DLBCL patients with MYD88^L265P^ and MYD88^other^ are likely to be two subgroups with different clinical and molecular characteristics. The survival of patients with MYD88^other^ is not superior than those with MYD88^L265P^, even poorer when focusing on the non-GCB subtype.

**Supplementary Information:**

The online version contains supplementary material available at 10.1007/s00432-023-04714-1.

## Introduction

Diffuse large B-cell lymphoma (DLBCL) is a highly heterogeneous lymphoid malignancy in adults, representing the most common entity in non-Hodgkin lymphomas (Alaggio et al. 2022). Despite about 60% DLBCL patients can be cured via standard front-line rituximab plus cyclophosphamide, doxorubicin, vincristine, and prednisone (R-CHOP), most of those refractory and relapse patients will succumb to their disease (Flowers et al. 2010; Shi et al. 2022). With the development of large-scale high-throughput sequencing technologies, the underlying heterogeneity in DLBCL has been characterized at an unprecedented scale. Genomic aberration-based classification of DLBCL subtypes is becoming increasingly accepted. Several taxonomies such as the four subtypes (MCD, N1, EZB, BN2), five subtypes (Cluster 1–5) or (MYD88, BCL2, SOCS1/SGK1, TET2/SGK1, and NOTCH2), and seven subtypes (MCD, N1, A53, BN2, ST2, EZB-MYC^+^, EZB-MYC^−^) have been reported (Schmitz et al. 2018; Chapuy et al. 2018; Lacy et al. 2020; Wright et al. 2020). Genetic subtypes provide useful prognostic information and treatment reference for clinical oncologist. Of special note is MYD88 mutation is a pivotal genetic driver determining the genetic classification in all above mentioned taxonomies and is mostly deemed to be an unfavorable prognostic factor along with CD79B mutation.

MYD88 encodes an adapter protein that functions as an essential signal transducer in the interleukin-1 and Toll-like receptor signaling pathways (Iwasaki and Medzhitov 2010; Ishii and Akira 2006). MYD88 Leu 265 Pro (L265P) mutation, occurred in about 30% of activated B-cell-like (ABC) DLBCL, is the most common non-synonymous and gain-of-function driver mutation. In contrast, this mutation is rare in GCB cases (Ngo et al. 2011; Dubois et al. 2017; Rovira et al. 2016a). Other recurrent non-L265P variants of MYD88 were also identified in DLBCL. However, their role in DLBCL lacks adequate attention due to the low prevalence. Several studies have indicated the difference between MYD88^L265^^P^ and MYD88^other^ but lacking of direct and detailed comparison in clinical and genetic features. Besides, the samples are limited, which might cause insufficient analysis and biased conclusions. Moreover, the survival of DLBCL patients with MYD88^L265P^ and MYD88^other^ varies in different studies, which is still a matter of debate (Chapuy et al. 2018; Dubois et al. 2017; Rovira et al. 2016; Xie et al. 2022).

Therefore, this study intends to investigate the clinical characteristics, genetic alteration, and survival of DLBCL patients between MYD88^L265^^P^ and MYD88^other^ variants through retrospective analysis of 132 patients with MYD88 variations in a cohort of 475 DLBCL patients from the Cancer Hospital, Chinese Academy of Medical Sciences & Peking Union Medical College (CHCAMS), and Suzhou Municipal Hospital, hoping to better detail the clinical and genetic background of DLBCL patients with MYD88^L265P^ and MYD88^other^.

## Materials and methods

### Patient selection and data collection

**Fig. 1:**
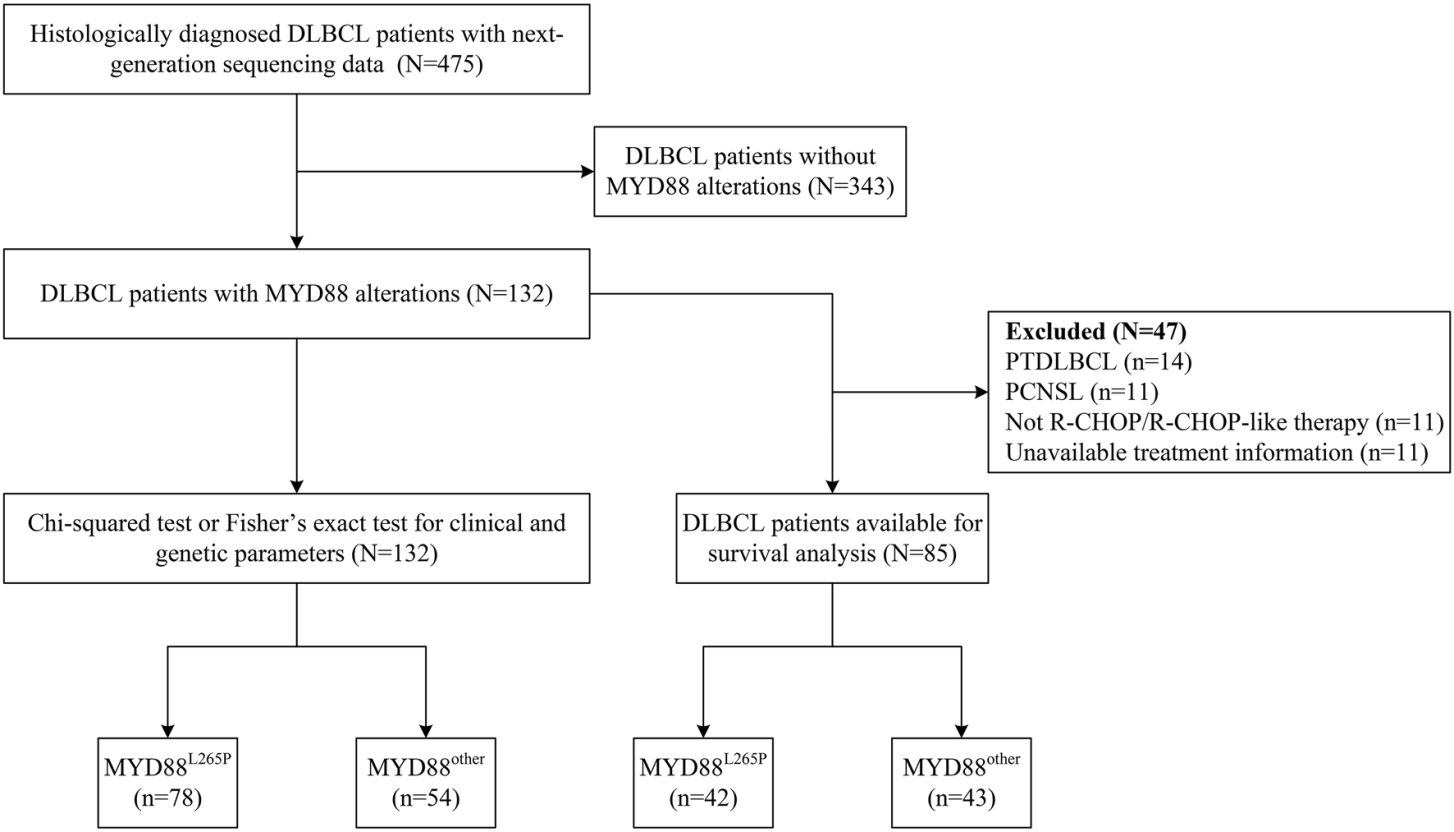
Flow chart of the study design

This study retrospectively collected 475 patients diagnosed with de novo DLBCL by two experienced pathologists at the CHCAMS and Suzhou Municipal Hospital from February 6th, 2007 to May 20th, 2022. A total of 132 patients with MYD88 variations were included for analysis. The inclusion criteria were as follows: (1) patients were histologically diagnosed with DLBCL based on the World Health Organization classification of Tumors of Hematopoietic and Lymphoid Tissue (2008) (Sabattini et al. 2010); (2) patients were confirmed MYD88 variation via next generation sequencing (NGS) technology as part of routine procedure; (3) patients who provided informed consent and adequate tissue for molecular and genetic detection. The exclusion criteria were those cases without adequate tissue for molecular and genetic detection or the information of MYD88 variation was unavailable. For survival analysis, we excluded patients with primary mediastinal large B-cell lymphoma, primary testicular DLBCL, and primary central nerves system lymphoma because of their particular biological behavior, clinical characteristics, treatment management, and prognosis. Only 85 patients who were received R-CHOP or R-CHOP-like regimens and with well-documented progression free survival (PFS) (from the date of initial diagnosis to the date of disease progression, relapse or death from any causes), overall survival (OS) (from the date of initial diagnosis to death from any causes), and survival status data were eventually included. The screening flowchart is displayed in Fig. 1. The study was approved by the Institutional Review Board of National Cancer Center/National Clinical Research Center for Cancer/Cancer Hospital, Chinese Academy of Medical Sciences & Peking Union Medical College (No. NCC2018JJJ-004) and was conducted in accordance with the ethical standards of the institutional committee and with the Declaration of Helsinki.

Patients baseline clinical characteristics, molecular and genetic alteration information, and follow-up data were collected, including gender, age, Ann Arbor stage, Eastern Cooperative Oncology Group (ECOG) performance status (PS), number of extranodal involvement sites, lactate dehydrogenase (LDH) level, International Prognostic Index (IPI) score, bulky disease, B symptoms, primary site, testicle/central nervous system (CNS) involvement, relapse, bone marrow/peripheral blood involvement, cell of origin (COO) type, first-line therapy, protein expression of CD5, CD10, CD20, BCL-2, BCL-6, c-MYC, MUM-1, Ki-67, and PD-L1, genetic alteration of CD79, TP53, BCL-2, BCL-6, c-MYC, PIM1, SGK1, cell cycle-related genes (CDKN2A, CCND3), NOTCH pathway-related genes (NOTCH1, NOTCH2, DTX1), JAK-STAT pathway-related genes (SOCS1, STAT3, STAT6), PI3K pathway-related genes (ITPKB, PTEN), immune-related genes (B2M, CIITA, CD58), epigenetics-related genes (KMT2D, KMT2C, CREBBP, EP300, TET2, EZH2, MEF2B), RAS pathway-related genes (GNA13, RHOA, RAS, BRAF). Any gene occurred alteration in the pathway will be defined as “pathway gene alteration”.

### Immunohistochemical staining and scoring

Formalin-fixed paraffin-embedded (FFPE) tissues were obtained from included patients. The protein expression of CD5, CD10, CD20, BCL-2, BCL-6, c-MYC, MUM-1, and Ki-67 were evaluated by immunohistochemistry (IHC) using associated antibodies (Fuzhou 100 Maixin Biotech, Fuzhou, China). The Programmed death-ligand 1 (PD-L1) expression was detected using the 22C3 anti-PD-L1 rabbit monoclonal antibody (Beijing Hightrust Diagnostic Company Limited, Beijing, China). The COO classification was confirmed according to the Hans algorithm (Hans et al. 2004). The expression of BCL-2 ≥ 60%, BCL-6 ≥ 30%, and c-MYC ≥ 40% of tumor cells was regarded as positive, respectively as previously reported (Zhang et al. 2018; Johnson et al. 2012).

### Detection of chromosome translocation

Interphase fluorescence in situ hybridization (FISH) was performed on three-micrometer-thick FFPE tumor tissues using Vysis LSI CMYC/BCL2/BCL6 Dual Color Break Apart Rearrangement Probe (Abbott Molecular, Abbott Park, IL, USA) according to the manufacturer’s instructions. Assessment of FISH signals was performed using Zeiss Axio Imager M2 epifluorescence microscope (Carl Zeiss, Oberkochen, Germany). Fifty tumor cells were counted, and the percentage of tumor cells with split-signal over 15% indicated the translocation of c-MYC, BCL-2, or BCL-6.

### Next-generation sequencing

Genomic DNA was extracted from the collected FFPE DLBCL tissues using the QIAamp DNA FFPE tissue kit (Qiagen, Hilden, Germany). DNA concentration was quantified using Qubit dsDNA HS Assay Kit (Invitrogen, Carlsbad, CA, USA). DNA fragmentation was conducted using Covaris S2 Ultrasonicator (Covaris, Woburn, MA, USA). Fragments of 200–250 bp were selected by AMPure beads (Agencourt AMPure XP kit; Beckman Coulter, Brea, CA, USA). End repair, phosphorylation, adaptor ligation, hybridization with capture-probe baits, hybrid selection with magnetic beads, and polymerase chain-reaction amplification were subsequently processed. Two capture panels were adopted, one consisted of 112 commonly altered genes in lymphoma and hematologic malignancies, the other covering 413 frequently mutated genes in DLBCL. There were 101 overlapping genes between the two panels. Capture-based targeted sequencing was performed on a Next Seq500 Sequencer (Illumina, Hayward, CA, USA) with pair-end reads at Geneplus-Beijing (Beijing, China) or Burning Rock Biotech (Guangzhou, China). Detailed sequencing procedure was performed as our previous study described (Qin et al. 2020, 2021; Jiang et al. 2020a, b).

### Statistical analysis

Chi-squared test or Fisher’s exact test when appropriated was adopted for the comparisons between categorical variables. Kaplan–Meier survival curve and log-rank test were performed for comparing the PFS and OS. *P*-value below 0.05 was considered statistically significant. All statistics were achieved via R software, version 4.21 (https://www.r-project.org/).

## Results

### Clinical and genetic characteristics of DLBCL patients with MYD88 variations

**Fig. 2:**
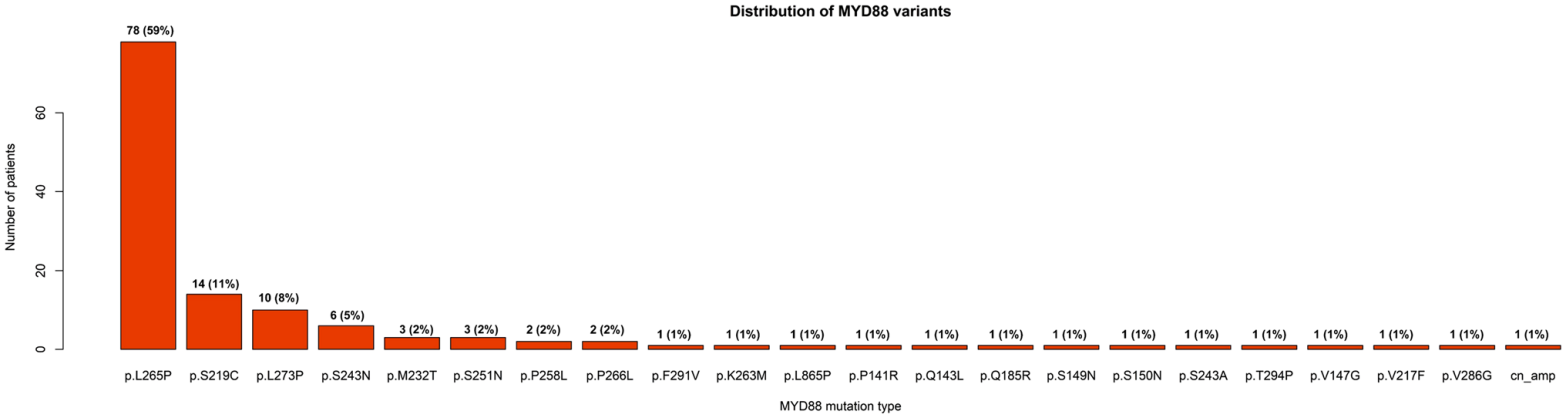
Distribution of MYD88 variants

**Fig. 3:**
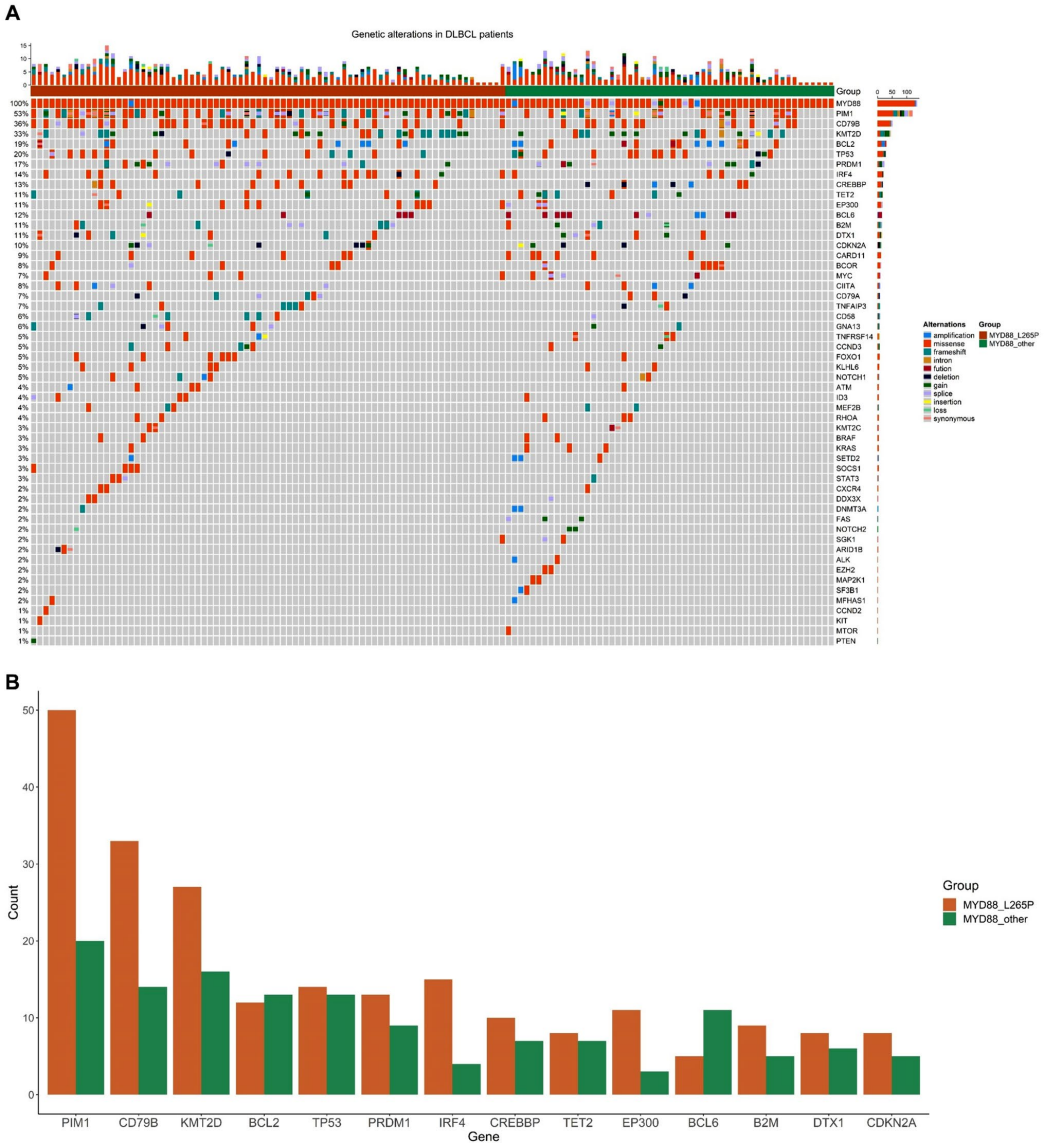
Genetic alterations in DLBCL. **A** Overview of the genetic alteration profile between MYD88^L265P^ and MYD88^other^. **B** Distribution of the top frequently mutated genes between MYD88^L265P^ and MYD88^other^

A total of 132 patients with MYD88 variations from a cohort of 475 DLBCL patients were included in this study. The variation rate of MYD88 is 28% (132/475). Among the patients with MYD88 variation, 59% (78/132) occurred MYD88^L265^^P^, while 41% (54/132) were MYD88^other^. Among patients with MYD88^other^, the p.S219C (14/54), p.L273P (10/54), and p.S243N (6/54) were the top three frequently occurred mutations (Fig. 2). Ninety-six percent (127/132) of the MYD88 variations were missense mutation. In addition to MYD88 variations, plenty of associated genes occurred genetic alterations in over ten percent of cases including PIM1 (53%), CD79B (36%), KMT2D (33%), TP53 (20%), BCL2 (19%), PRDM1 (17%), IRF4 (14%), CREBBP (13%), BCL6 (12%), BM2 (11%), TET2 (11%), EP300 (11%), and DTX1 (11%) (Fig. 3). The median age of the included patients was 62 years old. Patients diagnosed at limited stage (66/132) and advanced stage (63/132) were comparable, while the number of patients with IPI < 3 was almost twice than those patients with IPI ≥ 3. Most of patients were classified into non-GCB subtype (96/132). Twenty percent (27/132) of patients suffered testicle or CNS involvement. Seventy-four percent (98/132) of patients received R-CHOP or R-CHOP-like regimen. During the follow-up, forty-six percent (61/132) of the cases occurred disease relapse, while twenty-two percent (29/132) of the patients occurred death (Table 1).

**Table 1:** Patient characteristics according to MYD88 variation types

Variables	Total *n* (%)	MYD88^L265P^ *n* (%)	MYD88^other^ *n* (%)	*P*-value
COO subtype^a^				0.004
GCB	34 (26)	13 (17)	21 (39)	
Non-GCB	96 (73)	64 (82)	32 (59)	
Unknown	2 (1)	1 (1)	1 (2)	
Gender				0.133
Male	81 (61)	52 (67)	29 (54)	
Female	51 (39)	26 (33)	25 (46)	
Age^a^				0.662
Median age	62	62	63	
≥ 60	78 (59)	45 (58)	33 (61)	
< 60	52 (39)	32 (41)	20 (37)	
Unknown	2 (2)	1 (1)	1 (2)	
Ann Arbor stage^a^				0.044
I–II	66 (50)	45 (58)	21 (39)	
III–IV	63 (48)	32 (41)	31 (57)	
Unknown	3 (2)	1 (1)	2 (4)	
ECOG PS score^a^				0.752
≥ 2	21 (16)	13 (17)	8 (15)	
< 2	98 (74)	57 (73)	41 (76)	
Unknown	13 (10)	8 (10)	5 (9)	
Extranodal site^a^				0.044
≥ 2	46 (35)	22 (28)	24 (44)	
< 2	85 (64)	56 (72)	29 (54)	
Unknown	1 (1)	0 (0)	1 (2)	
LDH^a^				0.033
Elevated	51 (39)	24 (31)	27 (50)	
Normal	66 (50)	44 (56)	22 (41)	
Unknown	15 (11)	10 (13)	5 (9)	
IPI score^a^				0.161
≥ 3	39 (30)	19 (24)	20 (37)	
< 3	77 (58)	48 (62)	29 (54)	
Unknown	16 (12)	11 (14)	5 (9)	
Bulky disease^a^				0.182
Yes	21 (16)	10 (13)	11 (20)	
No	106 (80)	67 (86)	39 (72)	
Unknown	5 (4)	1 (1)	4 (8)	
B symptoms^a^				0.712
Yes	8 (6)	4 (5)	4 (7)	
No	120 (91)	73 (94)	47 (87)	
Unknown	4 (3)	1 (1)	3 (6)	
Lymph node/Lymphoid organ involvement only^a^				0.753
Yes	29 (22)	18 (23)	11 (20)	
No	102 (77)	60 (77)	42 (78)	
Unknown	1 (1)	0 (0)	1 (2)	
Testicle/CNS involvement^a^				0.000
Yes	27 (20)	24 (31)	3 (5)	
No	104 (79)	54 (69)	50 (93)	
Unknown	1 (1)	0 (0)	1 (2)	
Relapse^a^				0.058
Yes	61 (46)	31 (40)	30 (55)	
No	70 (53)	47 (60)	23 (43)	
Unknown	1 (1)	0 (0)	1 (2)	
Bone marrow/peripheral blood involvement^a^				0.685
Yes	6 (5)	3 (4)	3 (5)	
No	123 (93)	74 (95)	49 (91)	
Unknown	3 (2)	1 (1)	2 (4)	
Status				0.077
Alive	103 (78)	65 (83)	38 (70)	
Dead	29 (22)	13 (17)	16 (30)	
First-line therapy^a^				0.084
R-CHOP/R-CHOP-like	98 (74)	53 (68)	45 (83)	
Other therapy	20 (15)	15 (19)	5 (9)	
Unknown	14 (11)	10 (13)	4 (7)	
CD10 expression^a^				0.009
Positive	31 (24)	12 (15)	19 (35)	
Negative	98 (74)	64 (82)	34 (63)	
Unknown	3 (2)	2 (3)	1 (2)	
BCL-6 translocation				0.033
Yes	17 (13)	6 (8)	11 (20)	
No	115 (87)	72 (92)	43 (80)	
NOTCH pathway gene alteration				0.040
Yes	23 (17)	10 (13)	13 (24)	
No	109 (83)	68 (87)	41 (76)	
PIM1 mutation				0.000
Yes	76 (58)	55 (71)	21 (39)	
No	56 (42)	23 (29)	33 (61)	
PIM1 hypermutation				0.018
Yes	28 (21)	22 (28)	6 (11)	
No	104 (79)	56 (72)	48 (89)	

*COO* cell of origin, *GCB* germinal center B-cell, *Non-CGB* non-germinal center B-cell, *ECOG* Eastern Cooperative Oncology Group, *PS* performance status, *LDH* lactate dehydrogenase, *IPI* International Prognostic Index, *CNS* central nervous system

^a^*P*-values for the differences were calculated after excluding the unknown cases

### Clinicopathological characteristics and genetic alterations in different MYD88 variants

**Fig. 4:**
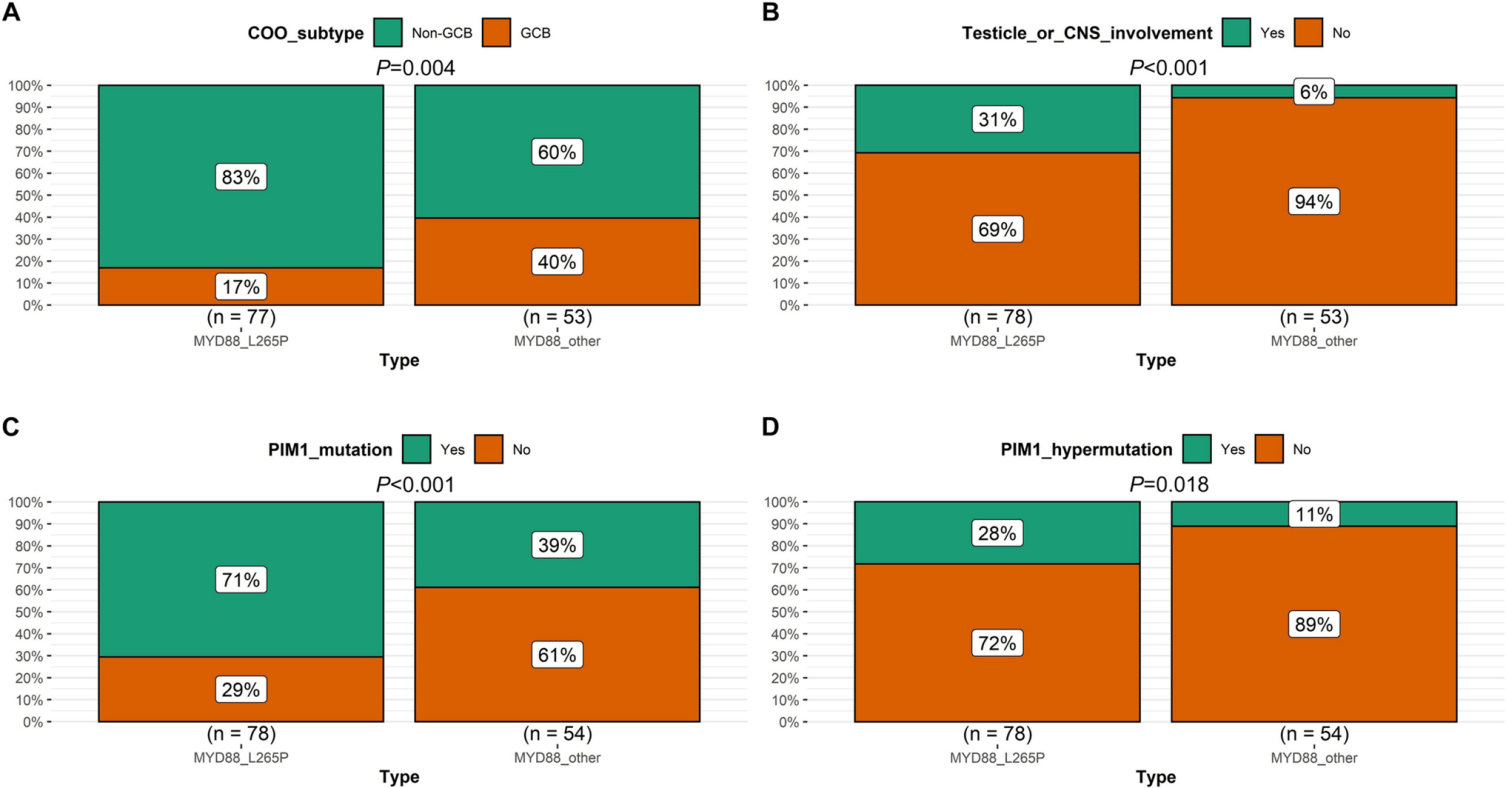
Significantly higher proportion of variables in MYD88^L265P^ than MYD88^other^. **A** COO subtype; **B** Testicle/CNS involvement; **C** PIM1 mutation; **D** PIM1 hypermutation

**Fig. 5:**
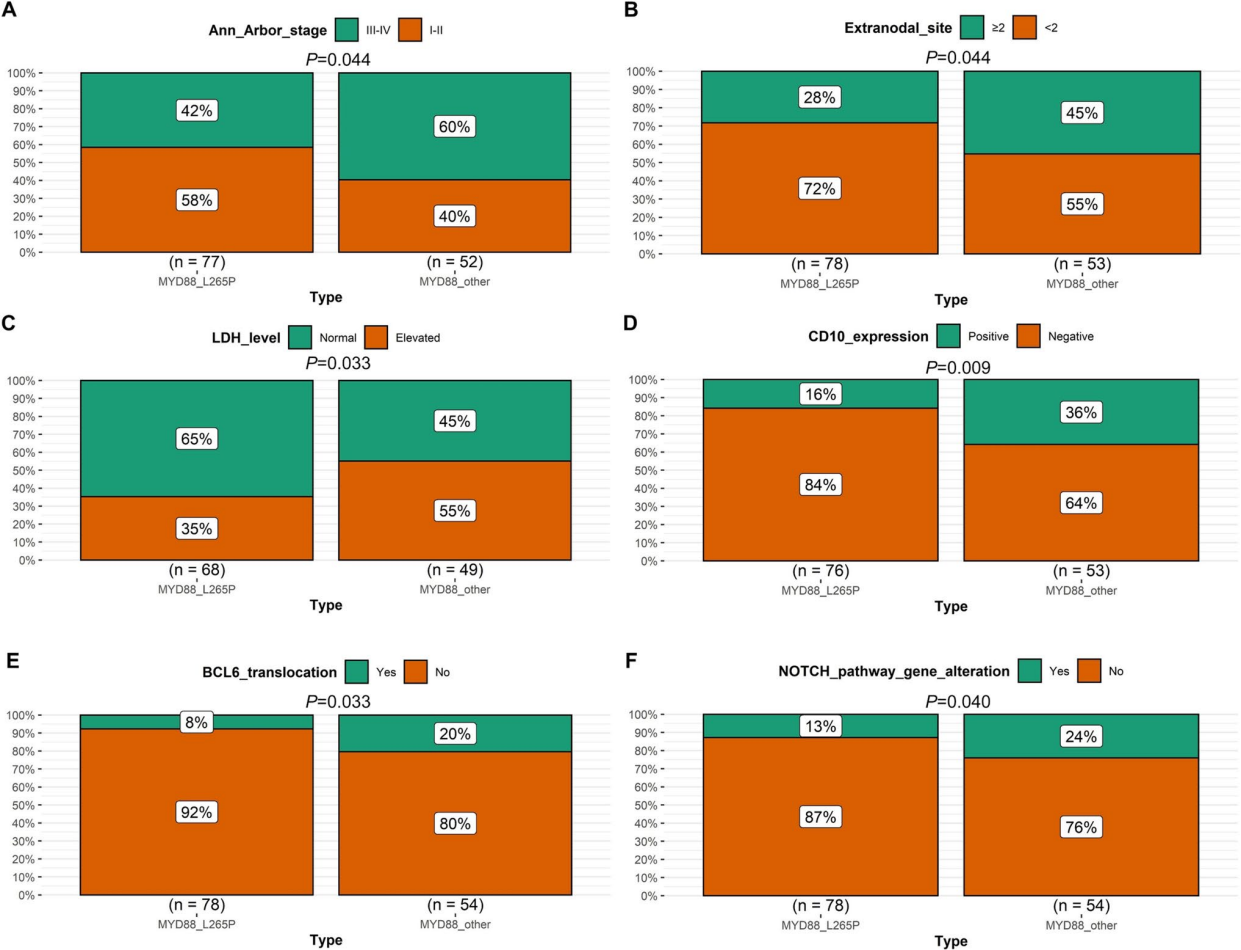
Significantly higher proportion of variables in MYD88^other^ than MYD88^L265P^. **A** Ann Arbor stage; **B** extranodal site; **C** LDH; **D** CD10 expression; **E** BCL-6 translocation; **F** NOTCH pathway gene alteration

Detailed patient clinicopathological characteristics and genetic alterations, as well as their associations with different MYD88 variation type were displayed in Table 1 and Supplementary file 1. We found that COO subtype, Ann Arbor stage, extranodal site, LDH, testicle/CNS involvement, CD10 expression, BCL-6 translocation, NOTCH pathway gene alteration, PIM1 mutation, and PIM1 hypermutation showed statistically significant distribution between MYD88^L265P^ and MYD88^other^ variants (Figs. 4 and 5). Specifically, MYD88^L265P^ was more common seen in non-GCB subtype than MYD88^other^ (83% vs. 60%, *P* = 0.004, Fig. 4A). Meanwhile, MYD88^L265P^ was significantly related to higher proportion of testicle/CNS involvement (31% vs. 6%, *P* < 0.001, Fig. 4B), PIM1 mutation (71% vs. 39%, *P* < 0.001, Fig. 4C), and PIM1 hypermutation (28% vs. 11%, *P* = 0.018, Fig. 4D), compared with MYD88^other^. Instead, MYD88^other^ were more likely to have higher percentage of advanced stage (60% vs. 42%, *P* = 0.044, Fig. 5A), extranodal site ≥ 2 (45% vs. 28%, *P* = 0.044, Fig. 5B), elevated LDH (55% vs. 35%, *P* = 0.033, Fig. 5C), positive CD10 expression (36% vs. 16%, *P* = 0.009, Fig. 5D), BCL-6 translocation (20% vs. 8%, *P* = 0.033, Fig. 5E), and NOTCH pathway gene alteration (24% vs. 13%, *P* = 0.040, Fig. 5F), compared with MYD88^L265P^.

### Treatment outcome between different MYD88 variants patients

**Fig. 6:**
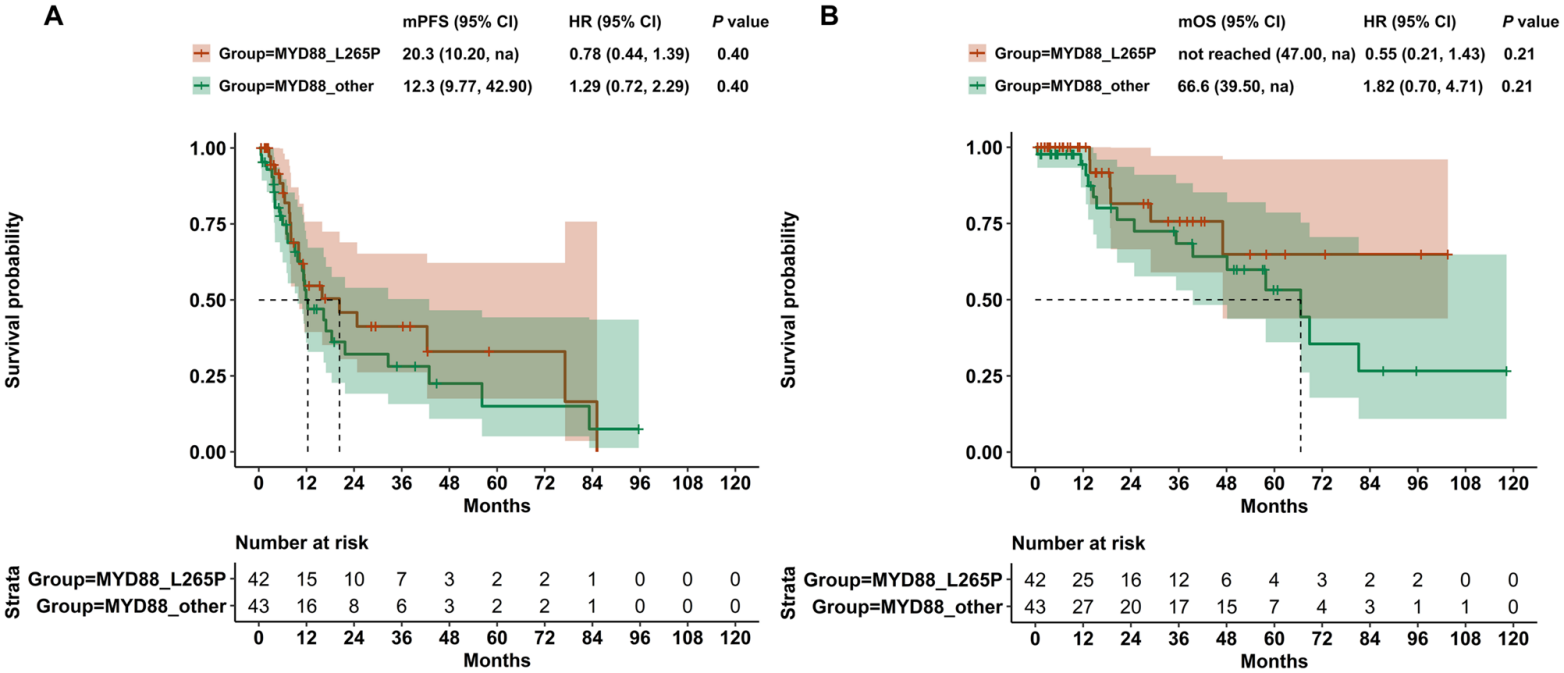
Prognostic significance of MYD88^L265P^ and MYD88^other^ in the whole DLBCL patients. **A** Progress-free survival. **B** Overall survival

A total of 85 patients were included for survival analysis, among which, 42 patients occurred MYD88^L265P^, while 43 patients occurred MYD88^other^. The median follow-up was 15.4 months (range: 0.5–118 months). A total of 21 patients eventually occurred death. No statistically significance was observed in PFS and OS between MYD88^L265P^and MYD88^other^ (Fig. 6).

**Fig. 7:**
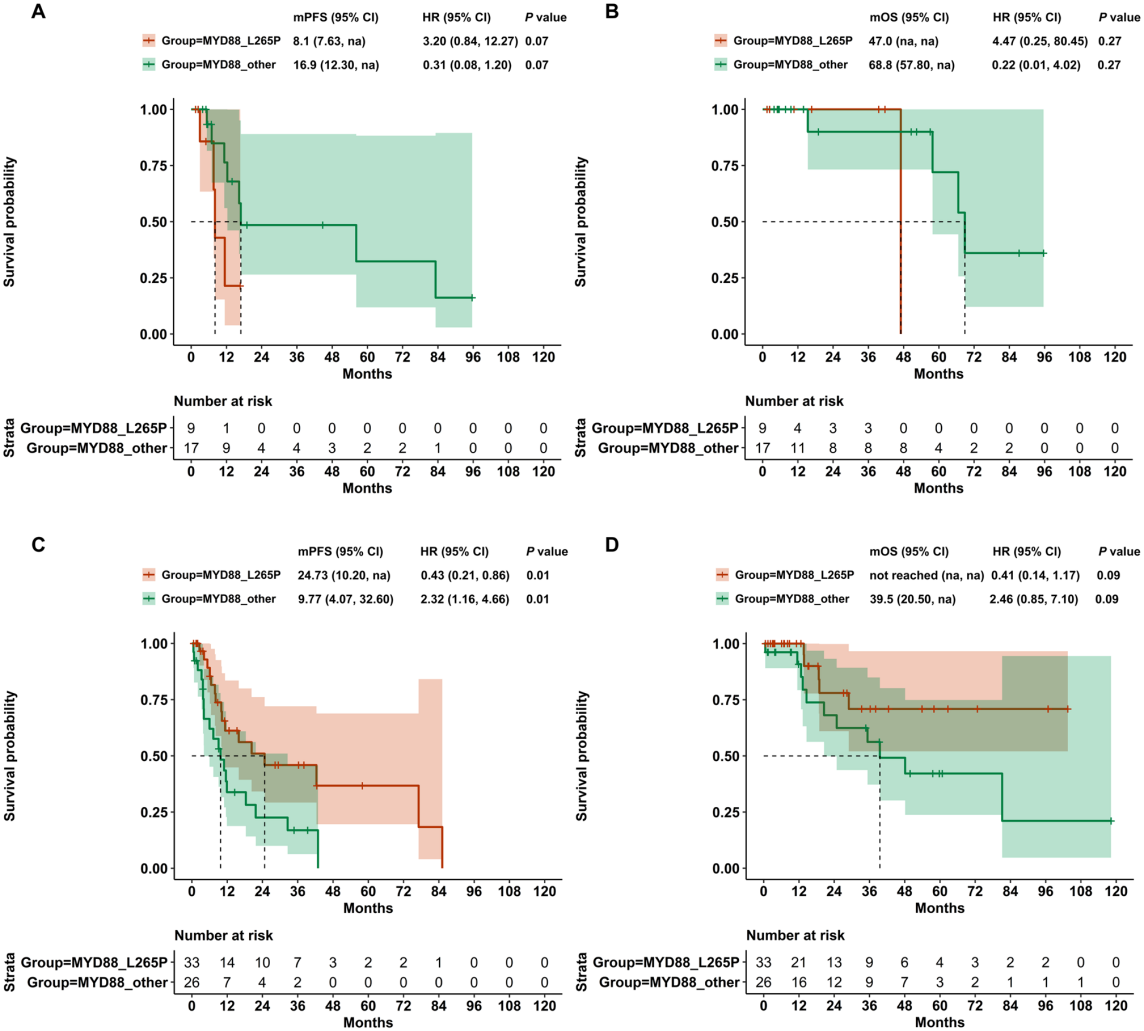
Prognostic significance of MYD88^L265P^ and MYD88^other^ in DLBCL subtypes. **A** Progress-free survival in GCB-DLBCL. **B** Overall survival in GCB-DLBCL. **C** Progress-free survival in non-GCB-DLBCL. **D** Overall survival in non-GCB-DLBCL

We further investigated the survival between MYD88^L265P^ and MYD88^other^ variants in GCB and non-GCB subtype. In non-GCB subtype, 33 patients occurred MYD88^L265P^, while 26 patients were MYD88^other^. Compared with MYD88^L265P^, patients with MYD88^other^ had worse PFS (median PFS: 9.77 months vs. 24.73 months, HR = 2.32, *P* = 0.010) (Fig. 7C). Regarding OS, patients with MYD88^other^ also showed a trend to suffer worse outcome than those with MYD88^L265P^ (median OS: 39.5 months vs. not reached, HR = 2.46, *P* = 0.090) (Fig. 7D). However, no statistical significance was observed for PFS or OS between MYD88^L265P^ and MYD88^other^ in GCB subtype (Fig. 7A, B).

## Discussion

MYD88 mutation is a widely accepted pivotal oncogenic driver in B-cell lymphomas, among which, the hotspot MYD88^L265P^ is mostly studied. In this study, the mutation frequency of MYD88^L265P^ is 59%, which is slightly lower than Dubois et al. reported as 64%. For MYD88^other^, Dubois et al. identified the frequent variants were p.S243A, p.S219C, p.V217P, and p.M232T, while our results showed that p.S219C, p.L273P, and p.S243N were the top three frequent variants (Dubois et al. 2017). This reflects great complexity and heterogeneity of MYD88^other^ variants in different cohorts of DLBCL. We found that MYD88^L265P^ was more common in non-GCB DLBCL compared with MYD88^other^ (83% vs. 60%, *P* = 0.004), which is consistent with previous reports (Ngo et al. 2011; Rovira et al. 2016a). As we know, the ABC DLBCL is characterized with chronic B-cell receptor signaling and activation of NF-κB (Davis et al. 2010, 2001; Havranek et al. 2017; Compagno et al. 2009). MYD88^L265P^ has been identified as non-synonymous and gain-of-function driver mutation, which can promote cell survival via assembling IRAK1 and IRAK4 contained protein complex, leading to IRAK4 kinase activity, IRAK1 phosphorylation, NF-κB signalling, and JAK kinase activation of STAT3 in ABC DLBCL (Ngo et al. 2011). This might help explain why MYD88^L265P^ is more frequent in non-GCB DLBCL. Nevertheless, in patients with MYD88^other^, non-GCB DLBCL still accounts for 60%, which might indicate the biological effect of some MYD88^other^ variants and MYD88^L265P^ is similar to some extent. A previous study also indicated their similarities. In Ngo’s study, most of MYD88^other^ reside in MYD88 Toll/IL-1 receptor (TIR) domain, which is the same domain as MYD88^L265P^ resides. As regards the ability to activate NF-κB, MYD88^wild−type^ presents modest activity, MYD88^L265P^ has strong activity, as does MYD88^M232T^ and MYD88^S243N^, while MYD88^S222R^ and MYD88^T294P^ exert intermediate effect. Their results suggested MYD88^other^ can contribute to the constitutive NF-κB activation in ABC DLBCL as with MYD88^L265P^ (Ngo et al. 2011). Given that, patients with MYD88^other^ should be also brought to attention in clinical. Intriguingly, there were 17% and 40% of GCB DLBCL patients with MYD88^L265P^ and MYD88^other^, respectively. Albeit GCB DLBCL is not typically characterized by constitutive NF-κB activation. However, classic and alterative NF-κB pathway can be activated in both ABC and GCB DLBCL. About 60% ABC DLBCL and 30% GCB DLBCL present nuclear localization of NFKB1/p50 (classical pathway) and NFKB2/p52 (alternative pathway) (Compagno et al. 2009). Besides, mutations of BCR/PI3K signaling intermediates (RHOA, GNA13, and SGK1) and NF-kB modifiers (CARD11, NFKBIE, and NFKBIA) were also found to be enriched in cluster 4 subtype of DLBCL, which is primary GCB DLBCL (Chapuy et al. 2018). Whether those GCB DLBCL with MYD88 alteration will be involved in NF-κB pathway remains to be explored in future study.

The genomic background hidden behind MYD88 is extremely complicated. We found that patients with MYD88 variation also frequently occurred PIM1, CD79B, KMT2D, TP53, BCL2, PRDM1, IRF4, CREBBP, BCL6, BM2, TET2, EP300, and DTX1 alterations. This is partly in accordance with previously reported results (Dubois et al. 2017; Shen et al. 2020). We found that the CD10 expression, NOTCH pathway gene alteration, and BCL-6 translocation were more common in MYD88^other^ than MYD88^L265^^P^. On the contrary, the PIM1 mutation and PIM1 hypermutation were more common in MYD88^L265P^ than MYD88^other^. These provided some clues implying why MYD88^other^ is more likely related to GCB subtype. For example, CD10 and BCL6 are markers of germinal center B cell. DLBCL patients with CD10 + , or CD10-/BCL6 + /MUM1- was classified into GCB subtype according to COO algorism (Alizadeh et al. 2000). Besides, according to several large-scale genetics-based classification of DLBCL subtype articles, PIM1 mutation was less frequent in GCB DLBCL (Schmitz et al. 2018; Chapuy et al. 2018; Lacy et al. 2020; Wright et al. 2020; Reddy et al. 2017). However, the BCL6 translocation, NOTCH1 mutation, and NOTCH2 mutation were all reported to be more common in non-GCB subgroup, which still remains confusing on their preference in MYD88^other (^Schmitz et al. 2018). Given above, the intricate genomic variation underlies MYD88 variation type and their relationship with COO subtype requires persistent investigation.

No significant difference in PFS and OS between MYD88^L265P^ and MYD88^other^ was observed in the whole DLBCL group. However, when focusing on the non-GCB type, the PFS of patients with MYD88^other^ was significantly shorter than those with MYD88^L265P^. Several studies have investigated the survival of DLBCL patients between MYD88^L265P^ and MYD88^other^ variants. In Dubois’s study, he performed a survival analysis on 26 ABC DLBCL patients with MYD88^L265P^ and 9 ABC DLBCL patients with MYD88^other^, who all received R-CHOP therapy. No statistical significance was found in OS and PFS (Dubois et al. 2017). Another study also investigated the PFS and OS between 39 patients with MYD88^L265P^ and 8 patients with MYD88^other^. Their results suggested that the OS and PFS of patients with MYD88^L265P^ showed a trend to be inferior than that of patients with MYD88^other^, but didn’t achieve statistically significant (Rovira et al. 2016). The samples in the two above studies are limited, which might lead to insufficient statistical efficacy and bias results. Compared with the above two studies, larger samples including 42 patients with MYD88^L265P^ and 43 patients with MYD88^other^ were performed for survival analysis in this study. MYD88^other^ was indicated as an unfavorable factor in DLBCL patients especially in non-GCB subgroup. Why patients with MYD88^other^ showed not superior survival than those with MYD88^L265P^ remains to be discussed. We did find that patients with MYD88^other^ presented higher percentage of advanced stage, extranodal site ≥ 2, and elevated LDH than those with MYD88^L265P^, which may provide some hints. Nevertheless, the genetic alteration and corresponding biological function difference between MYD88^L265P^ and MYD88^other^ may be the predominant factors influencing survival, which needs further study.

Altogether, this study highlights the clinical and genomic heterogeneity hidden behind the MYD88^L265P^ and MYD88^other^ variants. Furthermore, prognostic differences were revealed, most notably highlighting the survival of patients with MYD88^other^ is not superior than those with MYD88^L265P^, even poorer when focusing on the non-GCB subtype. Finally, this study added the importance of MYD88 variants status to the current knowledge and provided reference for the individualized targeted therapy and management of DLBCL patients.

## Supplementary Information

Below is the link to the electronic supplementary material.

Supplementary file1 (DOCX 23 KB)

## Data Availability

The data supporting the conclusions of this study can be obtained upon a reasonable request from the corresponding author.
