# Supplementary material for: MYD88L265P and MYD88other variants show different molecular characteristics and prognostic significance in diffuse large B-cell lymphoma
Source: J Cancer Res Clin Oncol. 2023 Apr 24;149(11):8483–94. doi: 10.1007/s00432-023-04714-1 (PMC10374827; doi:10.1007/s00432-023-04714-1)
Supplement: Supplementary file 1 — Supplementary file1 (DOCX 23 KB) [file 432_2023_4714_MOESM1_ESM.docx]

Supplementary file. Patient characteristics according to MYD88 variation types

| Variables | Total  n (%) | MYD88^L265P^  n (%) | MYD88^other^  n (%) | *P*-value |
| --- | --- | --- | --- | --- |
| CD5 expression^a^ |  |  |  | 0.920 |
| Positive | 15 (11) | 9 (12) | 6 (11) |  |
| Negative | 87 (66) | 51 (65) | 36 (67) |  |
| Unknown | 30 (23) | 18 (23) | 12 (22) |  |
| CD20 expression^a^ |  |  |  | 0.565 |
| Positive | 128 (97) | 77 (99) | 51 (94) |  |
| Negative | 3 (2) | 1 (1) | 2 (4) |  |
| Unknown | 1 (1) | 0 (0) | 1 (2) |  |
| BCL-2 expression^a^ |  |  |  | 0.334 |
| Positive | 73 (55) | 45 (58) | 28 (52) |  |
| Negative | 51 (39) | 27 (34) | 24 (44) |  |
| Unknown | 8 (6) | 6 (8) | 2 (4) |  |
| BCL-6 expression^a^ |  |  |  | 0.253 |
| Positive | 99 (75) | 56 (72) | 43 (80) |  |
| Negative | 23 (17) | 16 (20) | 7 (13) |  |
| Unknown | 10 (8) | 6 (8) | 4 (7) |  |
| C-MYC expression^a^ | |  |  | 0.505 |
| Positive | 42 (32) | 27 (35) | 15 (28) |  |
| Negative | 64 (48) | 37 (47) | 27 (50) |  |
| Unknown | 26 (20) | 14 (18) | 12 (22) |  |
| Double expressor^a^ |  |  |  | 0.161 |
| Positive | 28 (21) | 20 (26) | 8 (15) |  |
| Negative | 85 (65) | 48 (61) | 37 (68) |  |
| Unknown | 19 (14) | 10 (13) | 9 (17) |  |
| MUM-1 expression^a^ | |  |  | 0.286 |
| Positive | 113 (86) | 69 (88) | 44 (81) |  |
| Negative | 15 (11) | 7 (9) | 8 (15) |  |
| Unknown | 4 (3) | 2 (3) | 2 (4) |  |
| Ki-67 level^a^ |  |  |  | 0.448 |
| <60 | 11 (8) | 7 (9) | 4 (7) |  |
| 60-85 | 67 (51) | 37 (47) | 30 (56) |  |
| ≥85 | 51 (39) | 34 (44) | 17 (31) |  |
| Unknown | 3 (2) | 0 (0) | 3 (6) |  |
| PD-L1 expression^a^ |  |  |  | 0.302 |
| Positive | 65 (49) | 39 (50) | 26 (48) |  |
| Negative | 4 (3) | 1 (1) | 3 (6) |  |
| Unknown | 63 (48) | 38 (49) | 25 (46) |  |
| CD79A/B mutation | |  |  | 0.079 |
| Yes | 61 (46) | 41 (53) | 20 (37) |  |
| No | 71 (54) | 37 (47) | 34 (63) |  |
| TP53 mutation |  |  |  | 0.759 |
| Yes | 30 (23) | 17 (22) | 13 (24) |  |
| No | 102 (77) | 61 (78) | 41 (76) |  |
| BCL-2 translocation | |  |  | 0.158 |
| Yes | 5 (4) | 1 (1) | 4 (7) |  |
| No | 127 (96) | 77 (99) | 50 (93) |  |
| BCL-2 mutation |  |  |  | 0.581 |
| Yes | 17 (13) | 9 (12) | 8 (15) |  |
| No | 115 (87) | 69 (88) | 46 (85) |  |
| BCL-2 amplification | |  |  | 1.000 |
| Yes | 12 (9) | 7 (9) | 5 (9) |  |
| No | 120 (91) | 71 (91) | 49 (91) |  |
| BCL-6 mutation |  |  |  | 0.166 |
| Yes | 2 (2) | 0 (0) | 2 (4) |  |
| No | 130 (98) | 78 (100) | 52 (96) |  |
| BCL-6 amplification | |  |  | 0.359 |
| Yes | 3 (2) | 1 (1) | 2 (4) |  |
| No | 129 (98) | 77 (99) | 52 (96) |  |
| C-MYC translocation | |  |  | 0.716 |
| Yes | 8 (6) | 4 (5) | 4 (7) |  |
| No | 124 (94) | 74 (95) | 50 (93) |  |
| C-MYC mutation |  |  |  | 1.000 |
| Yes | 9 (7) | 5 (6) | 4 (7) |  |
| No | 123 (93) | 73 (94) | 50 (93) |  |
| C-MYC amplification | |  |  | 1.000 |
| Yes | 0 (0) | 0 (0) | 0 (0) |  |
| No | 132 (100) | 78 (100) | 54 (100) |  |
| Double/Triple hit |  |  |  | 0.166 |
| Yes | 2 (2) | 0 (0) | 2 (4) |  |
| No | 130 (98) | 78 (100) | 52 (96) |  |
| BCL-2/C-MYC double hit | |  |  | 0.409 |
| Yes | 1 (1) | 0 (0) | 1 (2) |  |
| No | 131 (99) | 78 (100) | 53 (98) |  |
| SGK1 mutation |  |  |  | 0.304 |
| Yes | 4 (3) | 1 (1) | 3 (6) |  |
| No | 128 (97) | 77 (99) | 51 (94) |  |
| Cell cycle gene alteration | |  |  | 0.727 |
| Yes | 25 (19) | 14 (18) | 11 (20) |  |
| No | 107 (81) | 64 (82) | 43 (80) |  |
| JAK-STAT pathway  gene alteration | | |  | 0.737 |
| Yes | 9 (7) | 6 (8) | 3 (6) |  |
| No | 123 (93) | 72 (92) | 51 (94) |  |
| PI3K pathway gene alteration | |  |  | 1.000 |
| Yes | 3 (2) | 2 (3) | 1 (2) |  |
| No | 129 (98) | 76 (97) | 53 (98) |  |
| Immune gene alteration | |  |  | 0.540 |
| Yes | 33 (25) | 21 (27) | 12 (22) |  |
| No | 99 (75) | 57 (73) | 42 (78) |  |
| Epigenetic gene alteration | |  |  | 0.177 |
| Yes | 80 (61) | 51 (65) | 29 (54) |  |
| No | 52 (39) | 27 (35) | 25 (46) |  |
| RAS pathway gene alteration | |  |  | 0.164 |
| Yes | 20 (15) | 9 (12) | 11 (20) |  |
| No | 112 (85) | 69 (88) | 43 (80) |  |

^a^ *P*-values for the differences were calculated after excluding the unknown cases.
